# Elemental Compositions of Enamel or Dentin in Human and Bovine Teeth Differ from Murine Teeth

**DOI:** 10.3390/ma16041514

**Published:** 2023-02-11

**Authors:** Steffen Möhring, Fabian Cieplik, Karl-Anton Hiller, Helga Ebensberger, Gerlinde Ferstl, Joshua Hermens, Melanie Zaparty, Ralph Witzgall, Ulrich Mansfeld, Wolfgang Buchalla, Konstantin Johannes Scholz

**Affiliations:** 1 Department of Conservative Dentistry and Periodontology, University Hospital Regensburg, Franz-Josef-Strauß-Allee 11, 93053 Regensburg, Germany; 2 Institute for Molecular and Cellular Anatomy, University of Regensburg, Universitätsstraße 31, 93053 Regensburg, Germany; 3 Bavarian Polymer Institute (BPI), University of Bayreuth, Universitätsstraße 30, 95447 Bayreuth, Germany

**Keywords:** enamel, dentin, elemental composition, murine, bovine, human, EDX, WDX

## Abstract

Teeth with different chemical compositions can show vastly different physical properties, so knowledge of elemental composition is required to use animal teeth as substitutes for human teeth in research. In vitro, energy dispersive X-ray spectroscopy (EDX), improved by calibration standards and Si_3_N_4_-window material, enables determining local elemental compositions of inorganic and organic compounds without sample destruction. Six human molars, bovine incisors, murine incisors, and murine molars were analyzed. EDX-field scans and line scans were analyzed for elements Ca, P, O, C, N, F, Na, Mg, Fe, Cl, and S. Furthermore, Ca/P- and Ca/N-Ratios were calculated. The presence of iron in murine incisor enamel was investigated using additional wavelength dispersive X-ray spectroscopy measurements (WDX) near the enamel surface. Bovine and human enamel and dentin revealed close similarities regarding elemental composition. The median (25–75% percentiles) of At%Ca was 21.1 (20.8–21.3) in human enamel, 21.0 (20.7–21.2) in bovine enamel, and in murine enamel, 18.3 (17.85–18.88) for molars and 18.35 (18.00–18.75) for incisors. In dentin, murine teeth revealed a higher At%Ca compared to human and bovine teeth. Significant differences between human and bovine teeth were found for nitrogen in dentin, with a median of 4.5 (3.3–5) At%N for human dentin and 2.7 (2.3–3.2) At%N for bovine dentin. The Ca/P-Ratio was the highest in human and bovine enamel, which did not differ significantly. Enamel from murine molars had a higher Ca/P-Ratio than murine incisors and the highest Ca/P-Ratio in dentin was observed for human teeth and murine molars (1.49). WDX revealed iron in the outer 10 µm of pre- and post-eruptive enamel of murine incisors. Pre- and post-eruptive enamel on murine incisors only differed significantly in At%Ca (*p* = 0.041) and At%P (*p* = 0.026) with both At% higher in the pre-eruptive enamel. Murine teeth differ significantly from human and bovine teeth in terms of the elemental composition of enamel and dentin.

## 1. Introduction

Murine and bovine teeth are commonly used in dental research, both in vitro and in situ [1,2,3,4]. While humans and cattle are diphyodont, mice, rats, and most other rodents are monophyodont with continuously growing incisors, showing pre- and post-eruptive areas [5]. A number of studies have investigated the similarities and differences between the properties of murine, bovine, and human teeth [3,6]. The micromorphology shows similarities in human and bovine dentin [7], while enamel crystallites in bovine teeth have larger diameters [8]. In murine teeth, the enamel prism patterns even differ between molars and incisors, with more variations regarding structural differences revealing radial and tangential (outer enamel) or lamellar (inner enamel) prism orientation in molars but only radial prism orientation in incisors [9,10]. Analysis of human and murine teeth revealed a similar dentin tubule diameter ranging between 1 and 1.6 µm [11]. Schmalz et al. found 32% smaller tubulus diameters in bovine compared to human dentin after using an aqueous solution of citric acid to expose the tubules [12].

Although it can be assumed, e.g., from studies investigating different areas of carious dentin, demineralized enamel, or patients with amelogenesis imperfecta [13,14,15], that the elemental composition influences the mechanical and chemical properties of dental hard tissues, none of the to date clinically applied diagnostic procedures aims for directly assessing the elemental compositions of dental hard tissues. In vivo, carious dental hard tissues are usually identified optically (scattering, discoloration), by probing (hardness), or by gray-scale analysis from dental film radiographs or microradiographs (density of atoms of different atomic numbers) [16,17,18]. Energy dispersive X-ray spectroscopy (EDX), although only available in vitro, provides an opportunity to locally determine elemental compositions without destroying the corresponding samples [19,20]. EDX systems of previous generations did not allow reliable detection of elements with atomic numbers below 10 [21]. More recent EDX systems with Si_3_N_4_ windows show a higher transmittance of X-rays compared to widespread polymer windows, especially for low keV X-rays emitted by elements of lower atomic numbers, and thus, allow detection of elements with atomic numbers < 10, such as nitrogen [20,22,23]. Accurate knowledge of structural properties and elemental composition is inevitably required to use extracted animal teeth as substitutes for human teeth for in vitro and in situ research [24]. In addition to elemental composition analysis, which includes spatial information assessed by EDX, Raman microscopy techniques may provide additional information about the presence of specific phosphate, carbonate, and organic structures [25]. By X-ray diffraction, information about the exact mineral composition (for example, the proportion of hydroxyapatite or carbonate-substituted hydroxyapatite) within dental hard tissues can be obtained [26]. However, when using these techniques, the teeth must be crushed and powdered, so in contrast to EDX, no direct local information or correlation with the morphology can be obtained.

The aim of the present study was to investigate the elemental composition of enamel or dentin from bovine, murine, and human teeth by means of energy-dispersive X-ray spectroscopy (EDX). The null hypothesis was that the elemental composition of human, bovine, and murine enamel or dentin does not differ significantly.

## 2. Materials and Methods

### 2.1. Specimens

#### 2.1.1. Murine Specimens

Six upper and lower jaws of three-month-old BL/6J mice held in the specific pathogen-free animal laboratory of the University of Regensburg were obtained from the dead animals, cleaned from soft tissues, and stored in 0.5% chloramine T solution (Merck KGaA, Darmstadt, Germany; pH 7.1). These mice originated from breedings for kidney research in which heterozygous transgenic mice were crossed with wildtype BL/6J individuals for more than twenty-five generations. As only heterozygous offspring could be used for further kidney research, siblings without transgenes were spare mice that had been euthanized without further processing the cadavers, so the teeth from the dead animals were used for our study.

After less than two weeks, one upper incisor and the largest first molar of each animal (4 molars from the upper and 2 molars from the lower jaw), not showing morphological irregularities were extracted and embedded in epoxy resin (EpoThin^TM^ 2, Buehler, Lake Bluff, IL, USA) enriched with graphite powder (West System, Bay City, USA; 7% concentration). During extraction, the incisors were marked just above the cervical bone margin by means of a scalpel (feather disposable scalpel, No. 15, Socorex, Ecublens, Switzerland) to differentiate between pre- and post-eruptive areas. The embedded specimens were ground from one approximal surface with FEPA P1200 on a water-cooled bench grinding machine (Metaserv Motopol 8, Buehler, Leinfelden-Echterdingen, Germany) with 3.6–4.0 relative centrifugal force (200 rpm) until the pulp was reached, resulting in half of the original tooth width for each specimen, and fined in three steps with FEPA P1500 and P4000 for 10 s per step.

#### 2.1.2. Bovine and Human Specimens

Six lower permanent incisors of freshly slaughtered bovine animals and six human caries free third molars were stored in 0.5% chloramine T solution (4 °C, pH 7.1) directly after extraction for a maximum of six months. Visual inspection and standard dental radiographs of bovine and human teeth were performed to identify and exclude teeth with caries.

The root was separated from the crown using a water-cooled cutting disc (Superdiaflex H 365F 190 Horico Dental, Berlin, Germany). Afterwards, the pulpal tissue was removed, and the crowns were cleaned of periodontal soft tissue remnants. The crowns were cut longitudinally in a vestibulo-oral direction into parallel slices (1000 µm thickness) with a saw microtome (Leitz 1600, Leica Microsystems, Wetzlar, Germany). The central slice was selected for examination. Furthermore, the surface located above the diamond saw blade and thus exposed to more water during cutting was polished manually in three steps with FEPA P1200, P1500, and P4000 for 60 s per step. The polished specimens were stored at 4 °C and 100% humidity and submitted to the subsequent analysis within a maximum of 48 h.

### 2.2. Scanning Electron Microscopy (SEM) and Energy Dispersive X-ray Spectroscopy (EDX)

**Figure 1:**
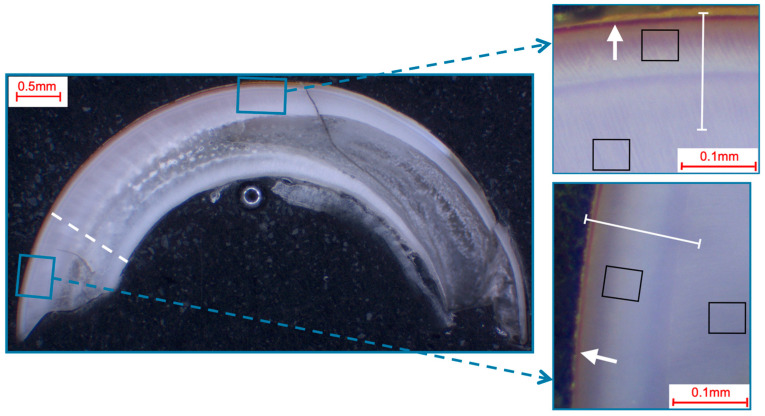
Selection of fields and line-scans for the elemental analysis in murine incisors shown in a photo-microscopic overview. The black squares indicate the 49 µm × 39 µm areas of the field scans, while the white lines indicate the paths of the line scans (length: approximately 140 µm). The white dotted line shows the pre/post-eruptive border, with the pre-eruptive scan area being enlarged in the top right and the post-eruptive scan area being enlarged in the bottom right picture. All pictures showed a yellow-brownish enamel, which is indicative of iron in the superficial enamel (white arrows).

**Figure 2:**
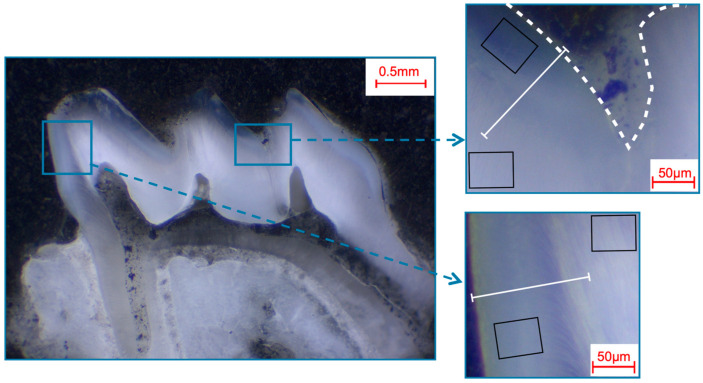
Selection of fields and line scans for the elemental analysis in murine first molars shown in a photo-microscopic overview. The white dashed line marks the enamel surface. The black squares indicate the areas of the 49 µm × 39 µm field scans, while the white lines indicate the approximately 140 µm paths of the line scans. The upper right picture shows the fissure area, while the bottom right picture shows the approximal area.

**Figure 3:**
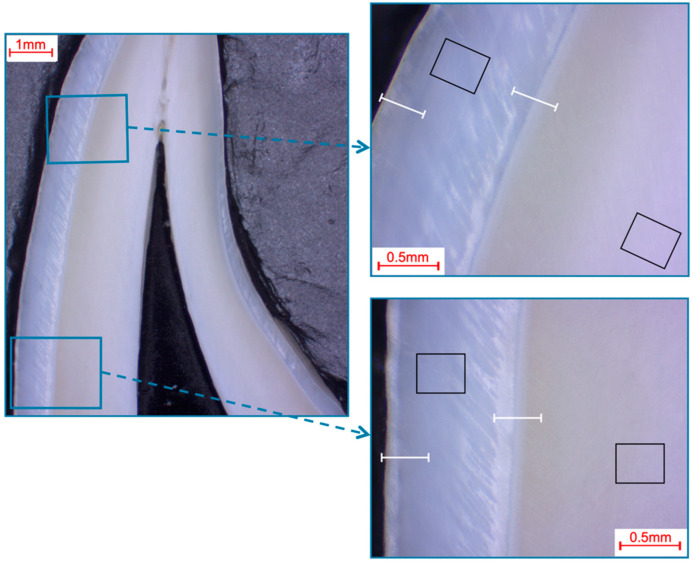
Selection of fields and line scans for the elemental analysis in bovine incisors shown in a photo-microscopic overview. The black rectangles indicate the areas of the 366 µm × 291 µm field scans, while the white lines indicate the approximately 360 µm paths of the line scans. The upper right picture shows the first measurement area (incisal crown), while the lower right picture shows the second measurement area (cervical crown), which was located halfway between the cemento-enamel junction and the first measurement area.

**Figure 4:**
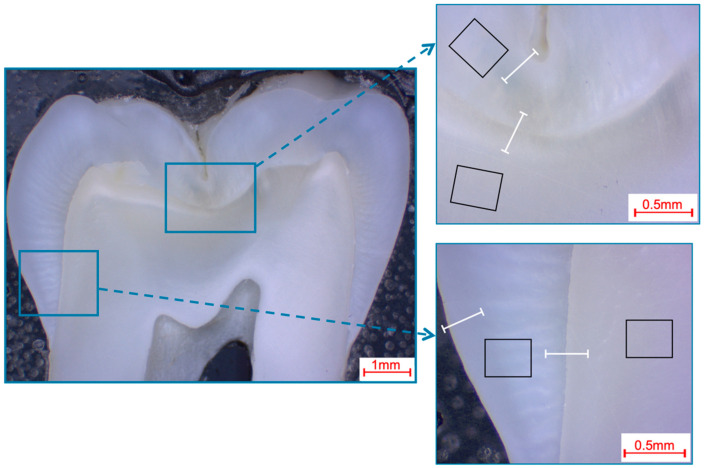
Selection of fields and line scans for the elemental analysis in human third molars shown in a photo-microscopic overview. The black rectangles indicate the areas of the 366 µm × 291 µm field scans, while the white lines indicate the approximately 360 µm paths of the line scans. The upper right picture shows the fissure area, while the bottom right picture shows the approximal area.

Photomicroscopic overview images were taken (M420, Wild, Heerbrugg, Germany; Axiocam 105 color, Carl Zeiss, Jena, Germany) to locate and visualize the areas to be analyzed (Figure 1, Figure 2, Figure 3 and Figure 4).

The specimens were mounted onto aluminum stubs (Baltic Präparation, e.K., Wetter, Germany) using double-sided adhesive carbon disks and conductive adhesive paste (Leit-C-Tab and Leit-C-Plast, Plano GmbH, Wetzlar, Germany). SEM-Overviews (horizontal field width of 340 µm for human and bovine samples; and of 140 µm for murine samples) for EDX field selection were made using low vacuum SEM (LV-SEM) without previous sputtering (FEI Quanta 400 FEG; Hillsboro, OR, USA; secondary electron mode, large field detector (LFD) and pressure limiting aperture (PLA); all components Thermo Fisher Scientific, FEI Deutschland GmbH, Frankfurt a. M., Germany; 1.5 Torr, accelerating voltage 6 kV, working distance 10 mm, pressure limiting aperture 500 μm, image resolution 2048 × 1768 pixels). A total of four EDX field measurements (two in enamel, two in dentin each in two different areas) and 2 EDX line scans per tooth were obtained.

The pre- and post-eruptive areas of the murine incisors were analyzed with the aid of the marker during extraction. In each area, enamel and dentin were first analyzed separately using 50 µm × 50 µm fields and additional exemplary 12 µm × 9 µm fields on the enamel surfaces of murine incisors. Furthermore, a line scan (0.2 µm resolution; ~700 measuring points, 140 µm approximate scan length) was drawn from the enamel surface into the dentin (Figure 1). On the murine molars, the approximal regions and the fissures were analyzed separately, and field measurements and line scans were performed according to the same parameters as on the murine incisors (Figure 2). On the bovine incisors, the first measurement area (incisal crown) was located on the height of the most incisal point of the crown pulp in the respective section. The second measurement area (cervical crown) was located halfway between the cemento-enamel junction and the first measurement field. One field (366 µm × 291 µm) was measured in the enamel and one in the dentin in each area for each specimen. In addition, line scans (0.9 µm resolution; ~400 measuring points; 360 µm approximate scan length) were performed from the surface into the enamel and from the enamel crossing the enamel-dentin junction into the dentin (Figure 3). Analogously to the murine molars, the approximal regions and the fissures were also defined as measurement areas for the human third molars. The same fields and line-scan dimensions were used as for the bovine anterior teeth (fields 366 µm × 291 µm; line scans 0.9 µm resolution, ~400 measuring points, 360 µm approximate scan length; Figure 4).

The relative surface elemental composition was measured using EDX, APEX, and EDS analysis systems (Octane Elect Plus detector, sw APEX v2.5; AMETEK GmbH, Meerbusch, Germany). The atomic percent (At%) of the elements F, C, N, Ca, P, O, Na, Mg, Fe, Cl, and S were recorded in this study. At%N and At%Ca were the target parameters of this experiment, representing the organic and inorganic portions of enamel or dentin. The EDX measurements were performed in low vacuum mode (1.5 Torr, accelerating voltage 6 kV for all specimens, and additional analyses using 15 kV on murine incisors aiming for the K_α1_-peak of iron, WD = 10 mm, aperture Ø 50 µm, measurement time 100 live seconds, image resolution 1024  ×  800 pixels).

### 2.3. Wavelength Dispersive X-ray Spectroscopy (WDX)

To reliably distinguish superficial iron from fluorine, additional WDX measurements were performed on one exemplary murine specimen using a Zeiss Ultra plus SEM equipped with a MagnaRay WDX detector (Thermo Fisher Scientific, Waltham, MA, USA) and an additional UltraDry EDX detector (Thermo Fisher Scientific) for region of interest selection. The sample was coated with 20 nm carbon prior to investigation using a Leica EM ACE 600 (Leica Microsystems). Analogously to the exemplary EDX scans, 10 µm × 10 µm fields of the enamel surface were analyzed. Additionally, a CaF_2_ and a fluorapatite standard (Geller Microanalytical Laboratory, Topsfield, MA, USA) were assessed.

### 2.4. Statistical Analysis

For EDX data, non-parametric statistical procedures were used to analyze the relative At% of respective elements (SPSS version 29, SPSS Inc., Chicago, IL, USA). Group medians, 25%- and 75%-percentiles from the representative values of the 6 specimens were determined for type of tooth and respective areas (murine incisor: pre- and post-eruptive; murine molar: approximal and fissure; bovine incisor: incisal crown and cervical crown; human molar: approximal and fissure) and dental hard tissues (enamel, dentin). In addition, results were summarized for the areas, as well as for all specimens regardless of species, type of tooth, or area. The dental hard tissues, enamel and dentin, were considered separately in all analyses. Ca/P- and, for dentin only, Ca/N-Ratios of corresponding At% values were calculated for the summarized types of teeth per species and dentin or enamel, respectively. Mann–Whitney *U*-tests were used to test for statistically significant differences between different groups and dental hard tissues. The level of significance was set to α = 0.05.

## 3. Results

**Figure 5:**
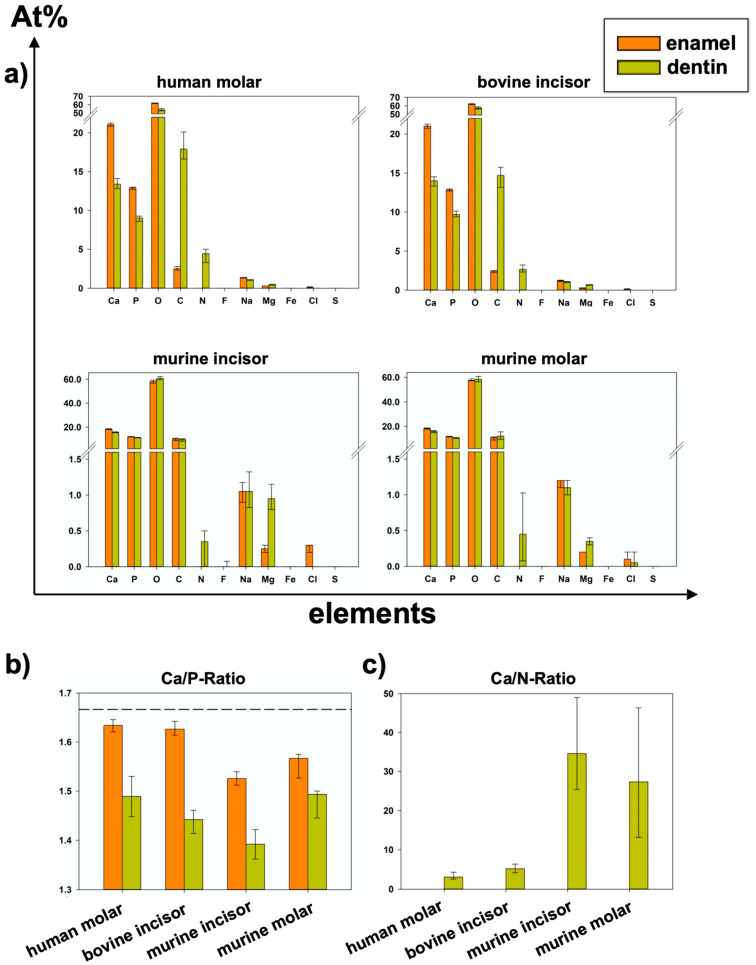
(Data also shown in Table 1; *p*-values in Table 2 and Table 4): (**a**) Elemental composition of summarized areas for each group of teeth separated by enamel or dentin (median and 25–75% percentiles). (**b**) Ca/P-Ratio of all specimens (median and 25–75% percentiles); dashed line shows stoichiometric ratio (1.667) of hydroxy apatite. (**c**) Ca/N-Ratio of all specimens (Median and 25–75% percentiles); Because At%N > 0 was only detected in dentin areas, Ca/N-Ratio could not be calculated for enamel.

**Figure 6:**
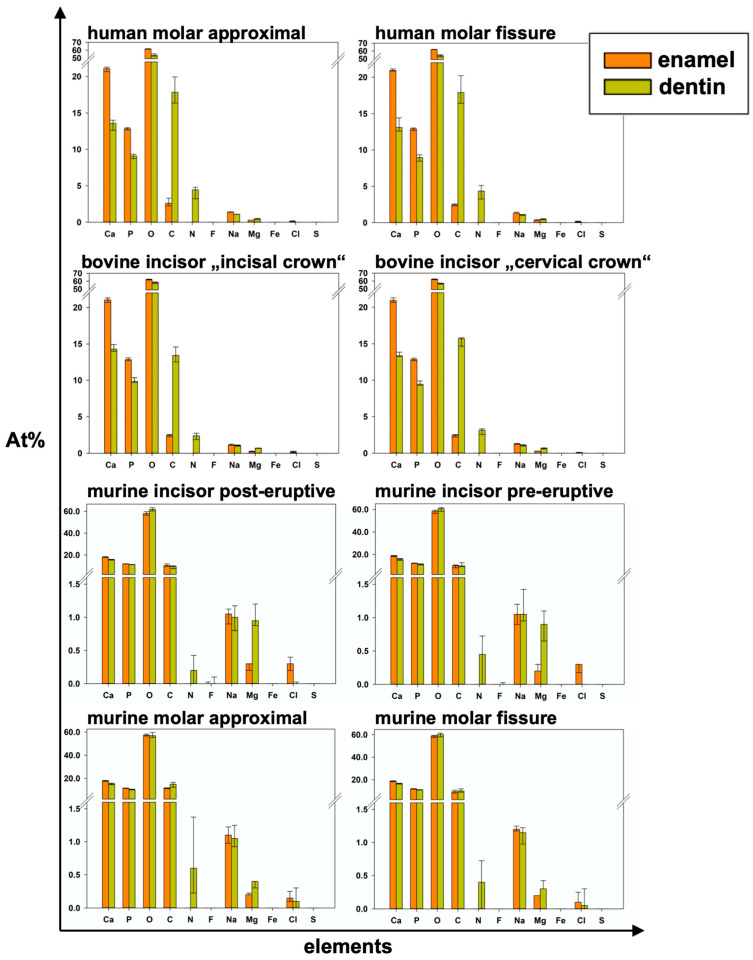
Elemental composition in detail for all areas and both examined dental hard tissues of each group and all elements (median and 25–75% percentiles); *p*-values in Table 3.

Table 1 and Figure 5a show the results of relative elemental composition analysis. Figure 6 illustrates the results of all eight analyzed areas separately. Significant differences between groups are shown in Table 2, Table 3, Table 4 and Table 5.

In all species, significantly higher At%Ca and At%P were found in the enamel than in the dentin of the specimens from the same group. Regarding At%P, there was a significant difference between the dentin of human molars and bovine incisors and between murine incisors and molars in both dental hard tissues. At%N was not detected in enamel specimens at all. Human dentin showed the highest At%N (4.45 At%N), followed by bovine, murine molars, and pre-eruptive and post-eruptive (0.2 At%N) dentin of murine incisors (Figure 6). At%O showed the highest relative value in all specimens. In human and bovine teeth, it was significantly higher in the enamel than in the dentin. Except for the post-eruptive area of murine incisors (*p* = 0.009), no significant differences in At%O were found in murine teeth between enamel and dentin. At%C was significantly higher in human and bovine dentin than in the corresponding enamel but showed no significant difference between the dentin and enamel in murine teeth.

**Figure 7:**
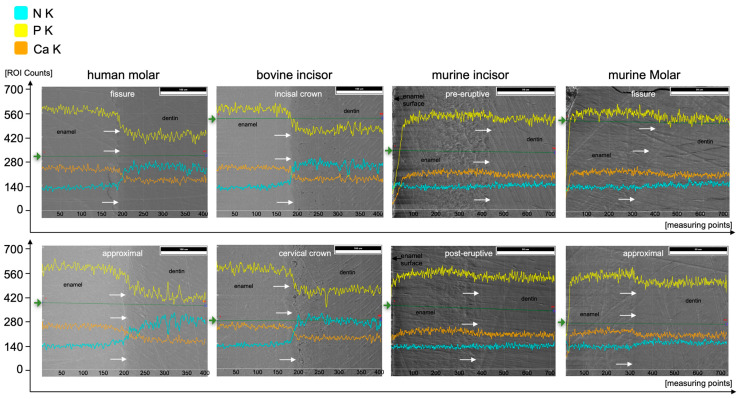
EDX line scans crossing the enamel-dentin junction (white arrows, enamel on the left, dentin on the right parts) of the respective species and areas along the green lines (starting from green arrows) for elements N (cyan), P (yellow), and Ca (orange).

At%Na was significantly higher in human enamel than in human dentin in the approximal (*p* = 0.002) and fissure areas (*p* = 0.002) and in bovine cervical enamel (*p* = 0.026). Human teeth showed the highest At%Na (1.4 At%Na in enamel) compared to bovine and murine teeth. Significantly more At%Mg was found in dentin than in enamel in all areas of all species (*p* ≤ 0.015). The Ca/P-Ratio was highest in human and bovine enamel, which did not differ significantly. Murine molars showed a higher Ca/P-Ratio than murine incisors in enamel and exhibited the highest Ca/P-Ratio in the dentin of all teeth in the study. The Ca/N-Ratio was also significantly (*p* ≤ 0.001) higher in the dentin of murine incisors (34.6) and molars (27.3) as compared to the other species (≤5.2) (Figure 5b and Table 1). Figure 7 shows EDX line scans of all groups crossing the enamel-dentin junction and showing more distinct differences between enamel and dentin in bovine and human teeth compared to murine teeth, particularly in calcium and nitrogen.

**Figure 8:**
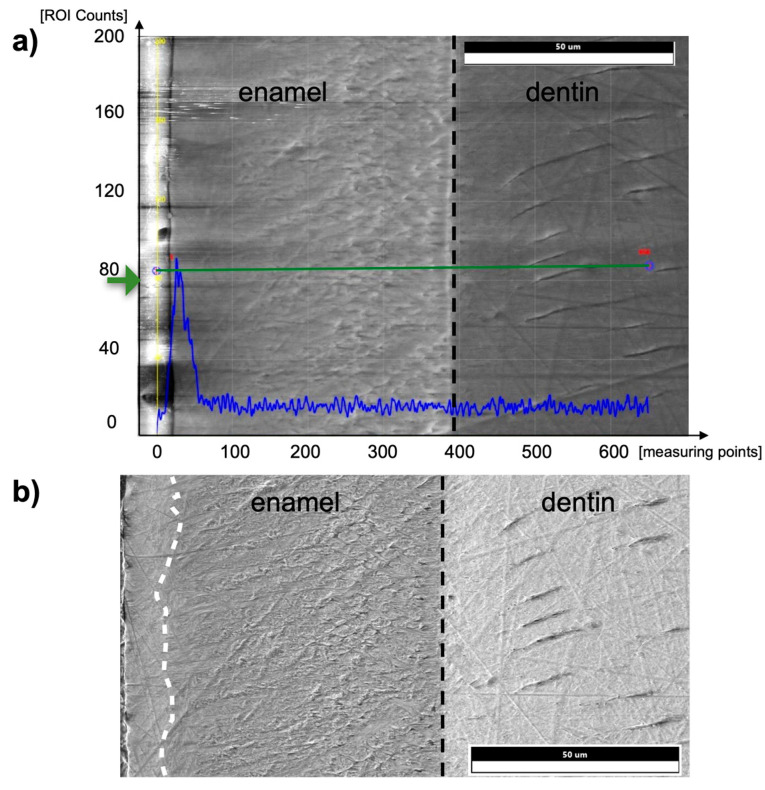
(**a**) EDX line scan from the tooth surface (left edge, enamel) into the dentin on a murine incisor in the post-eruptive area. The black dashed line indicates the enamel-dentin junction. The blue graph shows the counts in iron at the respective positions of the measuring line shown in green. (**b**) SEM image of a specimen of a murine incisor. The superficial 10 µm of enamel (left margin, separated by a white dashed line) shows a brighter texture than below the surface, and thus, a different micromorphology and presumably an aprismatic structure compared to the prismatic inner enamel.

Figure 8 shows an exemplary EDX line scan on the enamel surface and the enamel-dentin junction of a murine incisor in the post-eruptive area. Iron was detected in the outer 5–10 µm of the pre- and post-eruptive enamel of murine incisors. In addition, 9 µm × 12 µm field scans of the outer aprismatic enamel were performed on murine incisors, and Fe was found above the minimum detection limit with a median of 0.3 At%Fe ranging from 0 At%Fe to 0.5 At%Fe.

**Figure 9:**
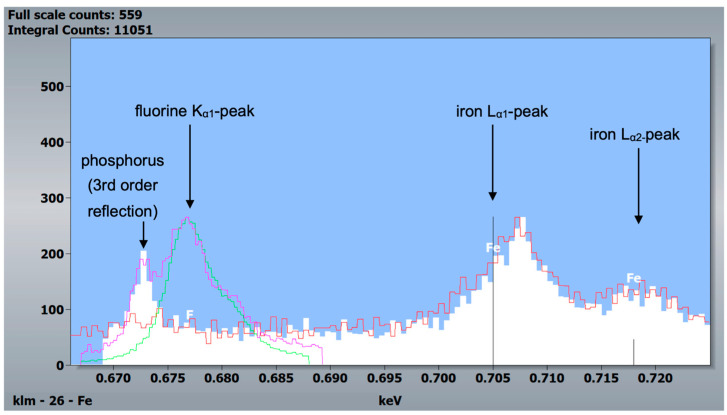
WDX measurements of a mouse specimen (post-eruptive enamel) and the two standards, CaF_2_ and fluorapatite. White and red: post-eruptive outer enamel, 10 kV and 5 kV accelerating voltage, respectively. Green: CaF_2_ standard, 20 kV accelerating voltage. Purple: fluorapatite standard, 20 kV accelerating voltage. Since the fluorine K_α1_-peak is at 0.677 keV and the iron L_α1_- and L_α2_-peaks are at 0.705 and 0.718 keV, respectively, this measurement indicates reliably that iron was detected in murine incisors and not fluorine.

Additional WDX scans of the same field revealed no fluorine but confirmed iron in the outer enamel layer (Figure 9).

## 4. Discussion

### 4.1. Discussion of Materials and Study Design

In the present study, enamel and dentin from teeth of three different species were compared regarding their elemental composition. In the literature, elemental compositions of teeth were analyzed mostly by grinding or milling specimens into particles, whereby spatial information is lost [27]. Using EDX, it was possible to determine specific fields on each specimen without processing the specimens into particles. In low vacuum mode (LV), the specimens could be measured directly after preparation without extensive exsiccation, as additional drying and sputtering were not necessary [23,28]. Due to the water vapor in the chamber, fewer exsiccation artifacts are expected, but a loss of resolution due to deflection of the electron beam by gas atoms in the SEM chamber (*skirt effect*) is also to be expected [23,29]. To reduce this *skirt effect* described for LV-EDX analyses, all our measurements were carried out using a pressure limiting aperture (PLA), which shortens the path of the electron beam through the chamber gas [30]. In this study, the generally applied accelerating voltage to obtain relative elemental compositions was 6 kV. This accelerating voltage was used because at least 1.5 times the X-ray emission energy of the electrons is required to excite the respective electrons for analysis [31]. To surely differentiate Fe (L_α1_-peak 0.705 keV) from F (K_α1_-peak 0.677 keV), additional field scans were performed on murine incisor enamel using an accelerating voltage of 15 kV, additionally aiming for the K_α1_-peak, which is present for iron (6.403 keV) but not for fluorine. Standard customized coefficient (SCC) factors aiming for improved analysis of elements with atomic numbers < 10 and, thus, lower energy X-ray lines, in particular nitrogen, were applied for all 6 kV measurements [22,23].

Although specimens were stored in chloramine T solution at neutral pH, At%Cl was 0 in most of the groups (Table 1) and above 0.1 At% by median only for the enamel of murine incisors (0.3 At%), so it is very unlikely that storing specimens in 0.5% chloramine T for up to 6 months lead to noteworthy contaminations or structural alterations that could affect the EDX measurements. Bovine incisors were included in the present study because they are used in dental research in numerous studies, for example, bond strength studies. In contrast, bovine premolars and molars are not suitable for the fabrication of multiple equal specimens because of their more complex geometry, whereas bovine incisors have been widely adopted as substitutes for all human tooth types [32]. In mice, the molars were included because they can be used within a caries model [33], and the incisors, because they erupt constantly, are therefore suitable for studying environmental influences on tooth development [34].

### 4.2. Discussion of the Results

Different studies applied EDX to analyze the composition of human enamel and dentin without or with structural abnormalities such as amelogenesis imperfecta or dentinogenesis imperfecta [13,35,36]. In contrast to the present study, some studies focused on single elements only without listing the results of all elements included, which, however, is crucial for a sound interpretation of the relative analyses [35,37]. The Ca/P-Ratio calculated from our results is slightly lower than the stoichiometric ratio of hydroxyapatite (1.667). Different studies using similar methods showed higher Ca/P-Ratios in enamel and dentin [35,38]. A reason for these differences could be found in the accelerating voltage used. Arnold and Gaengler used an accelerating voltage of 20 kV [35]. Since the K_α1_-peaks of calcium and phosphorus are 3.690 keV and 2.012 keV, respectively, an accelerating voltage of 20 kV can be considered too high. As mentioned above, an over-voltage range of at least 1.5 up to 3 is considered optimal [31], wherefore 6 kV would be the suitable accelerating voltage for the chosen experimental design to optimally excite and soundly analyze elements with an atomic number <10, such as nitrogen, on the one hand, but also cover the keV-range of calcium and phosphorus on the other hand. In addition, a Ca/N-Ratio was calculated to show the ratio between inorganic and organic components. Here, the nitrogen stands for the organic components, since it is contained in proteins and peptides. The lower nitrogen proportion in murine dentin subsequently resulted in a higher Ca/N-Ratio compared to the dentin of all other species. Here, the supposedly high variation, but at a very low level of relative At%N < 1 (Figure 5a) directly affects the Ca/N-Ratio with very high absolute variations (Figure 5c). Regarding At%Ca, differences between enamel and dentin were less distinct in murine teeth compared to human and bovine teeth (Figure 5b and Figure 7). The abrupt alterations at the enamel-dentin junction were also described for human and bovine teeth by other studies in the literature for human teeth [39].

Murine incisors may be advantageous for in vitro models because they exhibit pre-eruptive regions (without saliva exposure) and post-eruptive regions (with saliva exposure) with significant differences in enamel composition regarding At%Ca and At%O in phenotypically and genotypically healthy mice, as found in the present study. Other studies on human teeth indicate that longer-erupted enamel, presumably due to contact with saliva and fluorides, has a higher microhardness [40]. Thus, incisors can be used in the mouse model to separately investigate pre-eruptive structural abnormalities that have developmental causes and post-eruptive structural abnormalities that are related to their environment, e.g., nutrition, saliva, or bacteria. In contrast to mouse molars, incisors grow back continuously, which, on the one hand, could result in differences in amelogenesis between both tooth types and, on the other hand, allows investigation of environmental influences on amelogenesis as long as the mouse is alive.

Another difference between the murine incisors compared to murine molars, human molars, and bovine incisors was a detectable content of iron in the outermost 5–10 µm of enamel in the pre- and post-eruptive areas (Figure 8 and Figure 9). This enamel layer has an aprismatic structure and appears yellow-brownish in the photo-microscopic image (see Figure 1). Since the K_α1_-peak of fluorine is 0.677 keV and the L_α1_-peak of iron is 0.705 keV, we verified the iron peak by examining the K_α1_-peak of iron. Since this peak is at 6.398 keV and therefore cannot be excited with an accelerating voltage of 6 kV, 15 kV measurements were carried out on the murine incisors, and thereby the K_α1_-peak of the iron was observed. To be able to distinguish the iron L_α1_-peak from a possible fluorine K_α1_ -peak, WDX measurements were carried out on one exemplary specimen. As depicted in Figure 9, the X-ray energies of iron L_α1_ and fluorine K_α1_ could be separated into the WDX spectrum. At 5 kV (red), the post-eruptive outer enamel sample showed only the presence of iron, whereas no peak at the fluorine K_α1_ energy level was present. For comparison, the same sample was also analyzed at 10 kV (white), showing an additional peak arising at 0.672 keV, close to the expected fluorine energy of 0.677 keV. To clarify the origin of this peak, standard samples of CaF_2_ and fluorapatite, which both contain fluorine, were analyzed and showed a peak at 0.677 keV, matching the fluorine K_α1_ energy. Because no organic compounds were present, these standard samples were analyzed at 20 kV. In addition, the fluorapatite showed a peak at 0.672 keV, while the CaF_2_ did not. Thus, it can be concluded that the peak at 0.672 keV found in the teeth sample at 10 kV does not represent fluorine. In fact, the energy matches the 3rd order reflection of phosphorus (0.672 keV) present in the fluorapatite standard and in the tooth sample and can be considered an artifact intrinsic to the WDX detection. The reflection can be confirmed by increasing peak intensity at a higher acceleration voltage of 10 kV (Figure 9, white) compared to 5 kV (Figure 9, red) for the post-eruptive outer enamel. In this case, a reflection of a high X-ray energy would increase, while in contrast, a real X-ray at this low energy would decrease due to pronounced X-ray absorption. This is also the reason why 20 kV was chosen for the WDX of the standards. Furthermore, it is noticeable that the iron L_α1_-peak is shifted to higher energies (0.707 keV instead of 0.705 keV). A possible explanation for this finding is that iron is present in a higher oxidation state, such as a Fe^3+^ complex in oxidized hemoglobin, which can cause a shift to higher energies. This would also explain the yellow-brownish color of the enamel in the photo-microscopic images (Figure 1). On one hand, WDX provides better energy resolution compared to EDX, avoiding peak overlap, which is possible in this case. On the other hand, it can only be used in high vacuum mode and thus should just be used as an additional method for low-vacuum EDX when organic specimens are analyzed.

Since the iron was detectable in pre- and post-eruptive areas and the yellow-brownish color was visible in the entire enamel except for the most recently developed apical region, the incorporation of iron derived from hemoglobin before tooth eruption is a possible hypothesis. Further research will be necessary to determine the, to date, not fully understood origin and effect of iron in the enamel of murine incisors.

## 5. Conclusions

The null hypothesis was partially rejected by showing that there are significant differences in elemental composition between human and bovine teeth (mainly in dentin), but especially between murine compared to bovine or human enamel and dentin. Bovine enamel and dentin closely resembled corresponding human dental hard tissues regarding elemental composition. Murine teeth differed significantly from those of other species regarding enamel and dentin composition. Nevertheless, murine incisor teeth may be advantageous if pre- and post-eruptive enamel areas are needed for research focusing on environmental influences on amelogenesis.

## Figures and Tables

**Table 1:** Relative elemental composition (summarized areas) and Ca/P- and Ca/N-Ratios of all tooth types for enamel and dentin [At%], median and 25–75% percentiles of six specimens per group (data also shown in Figure 5 and Figure 6; *p*-values in Table 2 and Table 4). Elements Fe and S were measured but are not shown because the median and 25–75% percentiles were 0.00 At% in field scans.

			Ca	P	O	C	N	F	Na	Mg	Cl	Ca/P-Ratio	Ca/N-Ratio
**Human molar**	Enamel	**Median**	**21.05**	**12.90**	**61.80**	**2.50**	**0.00**	**0.00**	**1.40**	**0.30**	**0.10**	**1.63**	**–**
		25–75	20.83–21.25	12.70–13.00	61.43–62.05	2.33–2.78	0.00–0.00	0.00–0.00	1.30–1.40	0.30–0.30	0.10–0.20	1.62–1.65	–
	Dentin	**Median**	** 13.40 **	** 9.05 **	** 53.65 **	** 17.90 **	** 4.45 **	** 0.00 **	** 1.10 **	** 0.50 **	** 0.00 **	** 1.49 **	** 3.04 **
		25–75	12.80–14.13	8.58–9.28	52.73–55.25	16.63–20.10	3.30–5.00	0.00–0.00	1.03–1.10	0.40–0.50	0.00–0.00	1.45–1.53	2.53–4.28
**Bovine incisor**	Enamel	**Median**	** 21.00 **	** 12.85 **	** 62.20 **	** 2.45 **	** 0.00 **	** 0.00 **	** 1.20 **	** 0.30 **	** 0.10 **	** 1.63 **	** – **
		25–75	20.73–21.28	12.70–12.98	61.73–62.58	2.23–2.50	0.00–0.00	0.00–0.00	1.10–1.28	0.20–0.30	0.10–0.20	1.61–1.64	–
	Dentin	**Median**	** 14.00 **	** 9.70 **	** 57.15 **	** 14.70 **	** 2.65 **	** 0.00 **	** 1.05 **	** 0.70 **	** 0.00 **	** 1.44 **	** 5.16 **
		25–75	13.33–14.55	9.38–10.13	56.30–58.60	13.15–15.75	2.33–3.23	0.00–0.00	1.00–1.10	0.63–0.70	0.00–0.00	1.41–1.46	4.15–6.34
**Murine incisor**	Enamel	**Median**	** 18.35 **	** 12.00 **	** 58.25 **	** 10.25 **	** 0.00 **	** 0.00 **	** 1.05 **	** 0.25 **	** 0.30 **	** 1.53 **	** – **
		25–75	18.00–18.75	11.83–12.28	56.83–59.43	8.80–10.88	0.00–0.00	0.00–0.00	0.90–1.18	0.20–0.30	0.20–0.30	1.51–1.54	–
	Dentin	**Median**	** 15.75 **	** 11.35 **	** 60.80 **	** 9.70 **	** 0.35 **	** 0.00 **	** 1.05 **	** 0.95 **	** 0.00 **	** 1.39 **	** 34.60 **
		25–75	15.43–16.08	11.13–11.48	59.53–62.38	8.33–10.45	0.00–0.50	0.00–0.08	0.83–1.33	0.80–1.15	0.00–0.00	1.36–1.42	25.41–49.00
**Murine molar**	Enamel	**Median**	** 18.30 **	** 11.70 **	** 57.65 **	** 10.85 **	** 0.00 **	** 0.00 **	** 1.20 **	** 0.20 **	** 0.10 **	** 1.57 **	** – **
		25–75	17.85–18.88	11.50–12.00	57.18–58.85	9.03–11.60	0.00–0.00	0.00–0.00	1.10–1.20	0.20–0.20	0.10–0.20	1.53–1.57	–
	Dentin	**Median**	** 16.05 **	** 10.80 **	** 58.55 **	** 12.20 **	** 0.45 **	** 0.00 **	** 1.10 **	** 0.35 **	** 0.05 **	** 1.49 **	** 27.33 **
		25–75	14.95–16.73	10.33–11.00	56.45–60.90	9.63–15.68	0.08–1.03	0.00–0.00	1.00–1.20	0.30–0.40	0.00–0.20	1.45–1.50	13.17–46.38

**Table 2:** *p*-values from pairwise tests between enamel and dentin for the summarized areas in every group of teeth for all elements and the Ca/P-Ratio (results depicted in Figure 5).

Species, Tooth	Ca	P	O	C	N	F	Na	Mg	Fe	Cl	S	Ca/P-Ratio
**human molar**	<0.001	<0.001	<0.001	<0.001	<0.001	NS ^§^	<0.001	<0.001	NS	<0.001	NS	<0.001
**bovine incisor**	<0.001	<0.001	<0.001	<0.001	<0.001	NS	0.012	<0.001	NS	<0.001	NS	<0.001
**murine incisor**	<0.001	<0.001	0.001	NS	0.005	NS	NS	<0.001	NS	<0.001	NS	<0.001
**murine molar**	<0.001	<0.001	NS	NS	0.001	NS	NS	<0.001	NS	NS	NS	<0.001

^§^ NS, not significant (*p* > 0.05).

**Table 3:** *p*-values from pairwise tests between enamel and dentin in each measurement area for all elements and the Ca/P-Ratio (results in Figure 6).

Species, Tooth	area	Ca	P	O	C	N	F	Na	Mg	Fe	Cl	S	Ca/P-Ratio
**human molar**	**approximal**	0.002	0.002	0.002	0.002	0.002	NS ^§^	0.002	0.015	NS	0.002	NS	0.002
	**fissure**	0.002	0.002	0.002	0.002	0.002	NS	0.002	0.015	NS	0.002	NS	0.002
**bovine incisor**	**incisal crown**	0.002	0.002	0.002	0.002	0.002	NS	NS	0.002	NS	0.002	NS	0.002
	**cervical crown**	0.002	0.002	0.002	0.002	0.002	NS	0.026	0.002	NS	0.002	NS	0.002
**murine incisor**	**post-eruptive**	0.002	0.002	0.009	NS	NS	NS	NS	0.002	NS	0.002	NS	0.002
	**pre-eruptive**	0.002	0.002	NS	NS	NS	NS	NS	0.002	NS	0.002	NS	0.004
**murine molar**	**approximal**	0.002	0.002	NS	NS	0.015	NS	NS	0.004	NS	NS	NS	0.002
	**fissure**	0.002	0.002	NS	NS	NS	NS	NS	0.002	NS	NS	NS	0.009

^§^ NS, not significant (*p* > 0.05).

**Table 4:** *p*-values from pairwise tests between the groups of teeth for each dental hard tissue and all elements examined (summarized areas), as well as for the Ca/P- and Ca/N-Ratios (results in Figure 5 and Table 1).

Compared Species	Tissue	Ca	P	O	C	N	F	Na	Mg	Fe	Cl	S	Ca/P-Ratio	Ca/N-Ratio
**Human molar vs. bovine incisor**	**Enamel**	NS ^§^	NS	NS	NS	NS	NS	<0.001	NS	NS	NS	NS	NS	-
	**Dentin**	NS	<0.001	<0.001	<0.001	<0.001	NS	NS	<0.001	NS	NS	NS	0.005	<0.001
**Human molar vs. murine incisor**	**Enamel**	<0.001	<0.001	<0.001	<0.001	NS	NS	<0.001	0.014	NS	<0.001	NS	<0.001	-
	**Dentin**	<0.001	<0.001	<0.001	<0.001	<0.001	NS	NS	<0.001	NS	NS	NS	<0.001	<0.001
**Human molar vs. murine molar**	**Enamel**	<0.001	<0.001	<0.001	<0.001	NS	NS	<0.001	<0.001	NS	NS	NS	<0.001	-
	**Dentin**	<0.001	<0.001	<0.001	<0.001	<0.001	NS	NS	0.006	NS	0.039	NS	NS	<0.001
**Bovine incisor vs. murine Incisor**	**Enamel**	<0.001	<0.001	<0.001	<0.001	NS	NS	0.01	NS	NS	0.006	NS	<0.001	-
	**Dentin**	<0.001	<0.001	<0.001	<0.001	<0.001	NS	NS	<0.001	NS	NS	NS	0.006	<0.001
**Bovine incisor vs. murine molar**	**Enamel**	<0.001	<0.001	<0.001	<0.001	NS	NS	NS	0.014	NS	NS	NS	<0.001	-
	**Dentin**	<0.001	<0.001	NS	<0.001	<0.001	NS	NS	<0.001	NS	0.039	NS	0.007	<0.001
**murine incisor vs. murine molar**	**Enamel**	NS	0.017	NS	NS	NS	NS	NS	NS	NS	0.024	NS	0.007	-
	**Dentin**	NS	0.001	NS	NS	NS	NS	NS	<0.001	NS	NS	NS	<0.001	NS

^§^ NS, not significant (*p* > 0.05).

**Table 5:** *p*-values from pairwise tests between different measurement areas of the same dental hard tissues and the same type of teeth for all elements examined, as well as for the Ca/P- and Ca/N-Ratios (results depicted in Figure 6 and Table 1).

Compared Areas	Tissue	Ca	P	O	C	N	F	Na	Mg	Fe	Cl	S	Ca/P-Ratio	Ca/N-Ratio
**human approx. vs. fissure**	**Enamel**	NS ^§^	NS	NS	NS	NS	NS	NS	NS	NS	NS	NS	NS	-
	**Dentin**	NS	NS	NS	NS	NS	NS	NS	NS	NS	NS	NS	NS	NS
**bovine incisal crown vs. cervical crown**	**Enamel**	NS	NS	NS	NS	NS	NS	0.015	NS	NS	NS	NS	NS	-
	**Dentin**	0.041	NS	NS	0.026	0.026	NS	NS	NS	NS	NS	NS	NS	0.015
**murine incisor post- vs. pre-eruptive**	**Enamel**	0.041	0.026	NS	NS	NS	NS	NS	NS	NS	NS	NS	NS	-
	**Dentin**	NS	NS	NS	NS	NS	NS	NS	NS	NS	NS	NS	NS	NS
**murine molar approx. vs. fissure**	**Enamel**	0.041	NS	NS	0.009	NS	NS	NS	NS	NS	NS	NS	NS	-
	**Dentin**	0.015	0.009	NS	0.041	NS	NS	NS	NS	NS	NS	NS	NS	NS

^§^ NS, not significant (*p* > 0.05).

## Data Availability

All relevant data generated or analyzed during this study are included in this article. Further inquiries can be directed to the corresponding author.
